# Device-based measurement of physical activity in pre-schoolers: Comparison of machine learning and cut point methods

**DOI:** 10.1371/journal.pone.0266970

**Published:** 2022-04-13

**Authors:** Matthew N. Ahmadi, Stewart G. Trost

**Affiliations:** 1 Charles Perkins Centre, School of Health Sciences, Faculty of Medicine and Health, The University of Sydney, Sydney, NSW, Australia; 2 School of Human Movement and Nutrition Sciences, University of Queensland, St Lucia, QLD, Australia; 3 School of Exercise and Nutrition Sciences, Queensland University of Technology, Brisbane, QLD, Australia; Linneaus University, SWEDEN

## Abstract

**Introduction:**

Machine learning (ML) accelerometer data processing methods have potential to improve the accuracy of device-based assessments of physical activity (PA) in young children. Yet the uptake of ML methods by health researchers has been minimal and the use of cut-points (CP) continues to be the norm, despite evidence of significant misclassification error. The lack of studies demonstrating a relative advantage for ML approaches over CP methods maybe a key contributing factor.

**Purpose:**

The current study compared the accuracy of PA intensity predictions provided by ML classification models and previously published CPs for preschool-aged children.

**Methods:**

In a free-living study, 31 preschool-aged children (mean age = 4.0 ± 0.9 y) wore wrist and hip ActiGraph GT3X+ accelerometers while completing a video recorded 20-minute free play session. Ground truth PA intensity was coded continuously using the Children’s Activity Rating Scale (CARS). Accelerometer data was classified as sedentary (SED), light intensity (LPA), or moderate-to-vigorous intensity (MVPA) using ML random forest PA classifiers and published CPs for preschool-aged children. Performance differences were evaluated in a hold-out sample by comparing weighted kappa statistics, classification accuracy for each intensity band, and equivalence testing.

**Results:**

ML classification models (hip: κ = 0.76; wrist: κ = 0.72) exhibited significantly higher agreement with ground truth PA intensity than CP methods (hip: κ = 0.38–0.49; wrist: κ = 0.31–0.44). For the ML models, classification accuracy for SED and LPA ranged from 83% - 88%, while classification accuracy for MVPA ranged from 68% - 78%. For the CP’s, classification accuracy ranged from 50% - 94% for SED, 19% - 75% for LPA, and 44% - 76.1% for MVPA. ML classification models showed equivalence (within ± 0.5 SD) with directly observed time in SED, LPA, and MVPA. None of the CP’s exhibited evidence of equivalence.

**Conclusions:**

Under free living conditions, ML classification models for hip or wrist accelerometer data provide more accurate assessments of PA intensity in young children than CP methods. The results demonstrate the relative advantage of ML methods over threshold-based approaches and adds to a growing evidence base supporting the feasibility and accuracy of ML accelerometer data processing methods.

## Introduction

Regular physical activity during the early years is essential for healthy growth and development [1]. In recognition of the short- and long-term benefits of physical activity in young children, the World Health Organisation (WHO) has issued physical activity and movement guidelines for children from birth to 5 years [2]. Similar guidelines have been issued in the United Kingdom, Canada, and Australia [3–5]. Valid yet practical measures of physical activity for young children are needed, not only for assessing compliance with movement guidelines, but for evaluating the effectiveness of policies and practices to promote physical activity.

Accelerometer-based motion sensors are a well-established method for measuring physical activity in preschool-aged children [6, 7]. To date, most applications of accelerometry have involved the application of intensity-related thresholds or cut-points [8–10]. With this approach, the relationship between accelerometer output (i.e., proprietary activity counts or gravitational units) is established using linear regression and cut-points delineating established physical activity intensity categories are derived. Another common approach is to use of receiver operating characteristic (ROC) curves to identify cut-points that provide the best possible combination of sensitivity and specificity for differentiating adjacent physical activity intensity categories [11].

Although cut-points continue to be widely implemented there are significant limitations with this approach. The most notable limitation is that the relationship between accelerometer output and absolute energy expenditure varies considerably between activities and between individuals completing the same activity. The activity counts or gravitational units recorded by an accelerometer can be almost identical during activities with completely different energy expenditure. Conversely, accelerometer output can be completely different for activities with the same energy expenditure [9, 12]. Second, accelerometer output for a given activity is influenced by the participants age, stature, and motor competence [13]. Third, cut-point methods applied to activity counts or gravitational units recorded on the wrist can provide misleading estimates of physical activity intensity because they do not account for extraneous upper limb movements during sedentary activities or low intensity movements. Consequently, cut-points based on the relationship between accelerometer output and physical activity intensity are highly dependent on the child’s age and motor competence, accelerometer placement, and the activities included in the original calibration study. Not surprisingly, this has led to a proliferation of conflicting sets of intensity-related cut-points which exhibit significant misclassification error when tested in independent samples [14–17].

An alternative approach that has the potential to improve physical activity assessment among young children is machine learning. Machine learning is a form of artificial intelligence which mines accelerometer signal for patterns that can be used to make predictions of physical activity type and intensity [18]. In contrast to cut-point methods, which reduce the raw acceleration signal to a single value (i.e., activity counts), machine learning methods extract multiple "features" which describe the distributional and temporal dynamics of the accelerometer signal. These features serve as inputs to sophisticated statistical models or algorithms which predict physical activity type or energy expenditure.

When applied to children under five, as well as school-aged youth, machine learning approaches have been shown to provide accurate predictions of physical activity type and intensity [19–23]. However, to date, the uptake of machine learning methods by health researchers has been limited, primarily because of the need to process large quantities of raw accelerometer data using specialised software; and partly because of the lack of studies demonstrating a relative advantage of machine learning approaches over traditional cut-point methods. To date, only one study has compared machine learning techniques to traditional cut-point methods in preschool-aged children [24]. In that study, random forest and support vector machine physical activity classification models trained on features in the raw acceleration signal exhibited significantly better classification of physical activity intensity than activity count cut-points derived from the same training data. After mapping the five predicted activity classes to standard physical activity intensity categories, kappa statistics for the random forest and support vector machine models ranged from 0.76 to 0.84. In contrast, kappa for the cut-points ranged from 0.49 to 0.65. While these results supported the relative advantage of machine learning models over cut-point methods, the random forest and support vector machine models were trained on standardised activity trials, which are known to exhibit lower classification accuracy under true free-living conditions [25].

We recently trained and tested machine learned physical activity classification models for preschool-aged children using accelerometer data collected under true free-living conditions [19]. Classification accuracy for the best performing wrist and hip models was 80.6% and 85.9%, respectively. In comparison, accuracy for the laboratory trained wrist and hip classification models was 60.2% and 64.4%, respectively. However, the accuracy of the free-living machine learning classification models relative to traditional cut-point methods was not evaluated. Therefore, the purpose of this study was to compare the accuracy of physical activity intensity predictions provided by machine learning physical activity classification models for the hip and wrist, trained on free living data, to those provided by previously published cut-points for preschool-aged children under true free-living conditions.

## Materials and methods

### Participants

A total of 31 children between the ages of 3 and 5 years (mean age = 4.0 ± 0.9 y) participated in the study. Children were recruited through a university email list-serv, local media, and word of mouth. Twenty-one children were randomly assigned to the training sample to develop and evaluate the free-living machine learning classification models. The remaining 10 children served as a hold-out test sample to independently evaluate and compare the accuracy of the machine learning classification models and previously published cut-points for preschool-aged children. Prior to participation, parental written consent was obtained. The study was approved by the Queensland University of Technology’s Human Research Ethics Committee (Approval Number: 1700000423).

#### Free-living play session

Each child completed an active free play session at a location chosen by the parent or guardian. Sessions lasted approximately 20 minutes. Locations included the family home, community parks, and local green spaces. The research team provided age-appropriate toys and play equipment, and the children were free to engage in any activity they desired. This allowed for natural activity behavior, transitions, and engagement with peers and environment. A complete list of the physical activities performed during the play sessions and the median duration of each activity is provided in S1 Table in S1 File. During each session, a member of the research team used a Go-Pro Hero 5 (GoPro, Inc, San Mateo, CA) camera to video record participants for subsequent direct observation coding. Prior to the play session, an external timepiece was synchronized with the laptop computer used to initialize the accelerometers and displayed in front of the camera to ensure synchronization between Go-Pro video files and accelerometer timestamps.

During each free play session, children wore an ActiGraph GT3X+ accelerometer (ActiGraph Corporation; Pensacola, FL, USA) on their right hip and non-dominant wrist. For the hip location, the accelerometer was positioned on the right mid-axilla line at the level of the iliac crest. For the wrist location, the accelerometer was positioned on the posterior side of the arm, between the radial and ulnar styloid processes. The ActiGraph GT3X+ is a small and lightweight monitor that measures acceleration along three orthogonal axes with a dynamic range between +/- 6 g and a sampling frequency between 30–100 Hz. For the current study, the sampling frequency was set to 100 Hz.

### Direct observation and annotation procedures

Video files were imported into the Noldus Observer XT 14 software (Noldus Information Technology, Wageningen, the Netherlands) for continuous direct observation and coding. A customized two-stage direct observation scheme was implemented in which each movement event was first coded as one of five activity classes (sedentary = SED; light-intensity activities and games = L_ACT_G; moderate-to-vigorous intensity activities and games = M_ACT_G; walking = WALK; running = RUN) and then physical activity intensity using the Children’s Activity Rating Scale (CARS) [26]. The CARS coding scheme classifies activity into one of five categories 1 = stationary/motionless; 2 = stationary/movement of limbs or trunk (very easy); 3 = translocation (slow/easy); 4 = translocation (medium speed/moderate); 5 = translocation (fast or very fast/hard). If a participant was not in view of the camera, movement behavior was coded as "out of view". Inter-observer agreement was assessed by having two researchers independently code the movement events captured by five randomly selected videos. Cohen’s kappa coefficient for activity class was 0.91 (95% CI: 0.87–0.94) while kappa for activity intensity was 0.91 (95% CI: 0.90–0.92). The Observer XT software generated a vector of date-time stamps corresponding to the start and finish of each movement event. These date-time stamps were used to calculate event duration and assign an activity class and intensity code to the corresponding time segment of the accelerometer data.

### Accelerometer data processing and feature extraction

Following coding and annotation procedures, the accelerometer time series was converted into a single-dimension vector magnitude (VM) and segmented into 15 s windows (also known as epochs) for determination of the primary activity class, physical activity intensity and feature extraction. If the 15 s window contained multiple activity classes (e.g., a combination of MV_ACT_G and RUN), the final activity class assignment was the class that accounted for the majority of the window. For physical activity intensity, a weighted average CARS score was calculated by multiplying each numeric intensity code by the percentage of the 15 s window in that code and summing the products. Scores of less than 2.0 were categorized as sedentary (SED), while scores greater than or equal to 3.0 were categorized as moderate to vigorous physical activity (MVPA). Scores between 2.0 and 2.99 were categorized as light physical activity intensity (LPA). There is currently no consensus on the MET thresholds corresponding to SED, LPA, and MVPA in preschool-aged children. Butte and colleagues [27] proposed thresholds of 1.5 METs (SED/LPA), 2.8 METs (LPA/MPA), and 3.5 METs (MPA/VPA).

Twenty-five time and frequency domain features used in previously published physical activity classification models were extracted [20, 21, 25]. Feature included: mean, standard deviation, coefficient of variation, percentiles (10th, 25th, 50th, 75th, and 90th), skewness, kurtosis, maximum, minimum, peak to peak amplitude, median crossings, sum, mean absolute deviation, signal power, lag-1 autocorrelation, log energy, interquartile range, dominant frequency between 0.25 and 5.0 Hz, magnitude of dominant frequency between 0.25 and 5.0 Hz, and cross-correlation between each axis (xy, xz, and yz axes). A description of these features and associated descriptive statistics are provided in S2 Table in S1 File.

### Training and evaluation of machine learning models

The supervised learning algorithm used to develop the physical activity classification models was a random forest. A random forest is an ensemble of decision tree models. Each tree is learned on a bootstrap sample of training data, and each node in the tree is split using a randomly selected sample of features. The decisions from each tree are aggregated, and a final model prediction is based on majority vote. We elected to train random forest models because our previous work has shown them to provide more accurate predictions than other widely implemented supervised learning algorithms such as decision trees and support vector machines [21, 24]. Moreover, random forest models require very little pre-processing of the data, as the features do not need to be normalized and feature selection procedures are not required because the algorithm effectively does this on its own. Additionally, the model is resistant to over fitting the training data because each tree within the forest is independently grown to maximum depth using a randomly selected subset of features [28].

Random forest models were trained and cross-validated using the "randomForest", and "caret" packages within R software [29, 30]. The number of features randomly sampled at each node was set at three whilst the number of trees generated was set to 500. Predictive accuracy was evaluated using leave-one-subject-out cross-validation (LOSO-CV). In LOSO-CV, the classification model is trained on data from all the participants except one, which is "held out" and used as the test data-set. The process is repeated until all participants have served as the test data-set, and the performance evaluation results are averaged. Model performance was evaluated in terms of overall classification accuracy, calculated as the percentage of 15 s time windows correctly classified. In addition, confusion matrices were generated summarize classification accuracy for each activity class and identify patterns of misclassification.

### Comparison to cut-point methods

Raw accelerometer signal was converted to ActiGraph proprietary activity counts or gravitational unit metrics (Euclidean norm minus one = ENMO; Signal vector magnitude gravity = SVM *g*), and classified as SED, LPA, and MVPA using the thresholds reported in previous studies. In total, eight sets of cut-points developed for preschool-aged children were compared: four for the hip placement and four for the wrist placement. A description of each cut-point used in the study is provided in Table 1. For these comparisons, the five activity classes predicted by the physical activity classification models were mapped onto the traditional three intensity categories (SED, LPA, and MVPA) by collapsing the M_ACT_G, walking, and running activity classes into a single MVPA category. The sedentary and L_ACT_G classes were mapped to the SED and LPA categories, respectively.

**Table 1:** Preschool hip and wrist accelerometer cut points.

Location	Author	Sample	Unit	Cut points
Hip	Pate [31]	N = 29	Vertical axis	SED ≤ 200
		Age Range = 3–6 y	activity counts	LPA > 200
		Mean age = 4.4 y		MVPA ≥ 420
		13 boys, 16 girls		
	Evenson [32]	N = 33	Vertical axis	SED ≤ 25
		Range = 5–9 y	activity counts	LPA > 25
		Mean age = 7.3 y		MVPA ≥ 574
		12 boys, 21 girls		
	Butte [27]	N = 50	Vector magnitude	SED ≤ 205
		Range = 3–6 y	activity counts	LPA > 205
		Mean age = 4.5 y		MVPA ≥ 977
		25 boys, 25 girls		
	Crotti [33]	N = 32	ENMO	SED < 20 m*g*
		Range = 5–7 y		LPA ≥ 20 m*g*
		Mean age = 6.4 y		MVPA ≥ 95 m*g*
		15 boys, 17 girls		
Wrist	Johansson [34]	N = 26	Vertical axis	SED ≤ 267
		Range = 1–3 y	activity counts	LPA > 267
		Mean age = 2.2 y		MVPA ≥ 1320
		16 boys, 10 girls		
	Li [35]	N = 34	Vector magnitude	SED ≤ 639
		Range = 3–5 y	activity counts	LPA > 639
		Mean age = 4.0 y		MVPA ≥ 1765.75
		14 boys, 20 girls		
	Roscoe [36]	N = 21	SVM g*	SED < 60.6 m*g*
		Range = 4–5 y		LPA ≥ 60.6 m*g*
		Mean age = 4.7 y		MVPA ≥ 98.3 m*g*
		13 boys, 8 girls		
	Crotti [33]	N = 32	ENMO	SED < 36 m*g*
		Range = 5–7 y		LPA ≥ 36 m*g*
		Mean age = 6.4 y		MVPA ≥ 189 m*g*
		15 boys, 17 girls		

* Roscoe cut-points were scaled to frequency independent values by dividing the cut points by the sampling frequency (87.5hz) and window size (1s) used in the original publication; ENMO = Euclidean Norm Minus One; SVM = Signal Magnitude Vector; m*g* = Milligravity units; All cut-points are shown for values of 15s windows for comparison purposes.

### Statistical analysis

Classification accuracy for the machine learning classification models and cut-points were evaluated using weighted Kappa statistics. For the interpretation of Kappa, we followed the ratings suggested by Landis and Koch [37]: poor (0.0–0.2), fair (0.2–0.4), moderate (0.4–0.6), substantial (0.6–0.8), and almost perfect (0.8–1.0). Kappa statistics with non-overlapping 95% confidence intervals were deemed to be different at the 5% level of significance. In addition, heat map confusion matrices were generated to summarize classification accuracy in each intensity category and identify patterns of misclassification. The equivalence of observed and predicted time spent in SED, LPA, and MVPA for the entire play session was evaluated using 95% equivalence testing [38]. To reject the null hypothesis of the equivalence test, the 90% confidence interval for the difference was required to fall entirely within the prespecified equivalence bounds. Equivalence bounds were set at ± 0.5 standard deviations for directly observed time in SED, LPA, and MVPA [39]. Equivalence testing procedures were performed using the "TOSTER" package in R [40] and weighted Kappa coefficients were computed using the "DescTools" package in R [41].

## Results

### Cross-validation of machine learning models

Overall accuracy for the hip and wrist classifiers was 81.1% (95% CI: 79.1% - 83.0%). And 75.1% (95% CI: 72.8% - 77.2%), respectively. The confusion matrix is shown in Table 2. For both placements, classification accuracy exceeded 75% for sedentary behaviours and 80% for light-intensity activities and games. Almost all the sedentary misclassification (16.9% - 21.8%) occurred as light-intensity activities and games, whilst the majority of misclassification for light-intensity activities and games (6.5% - 6.9%) occurred as walking. Recognition of moderate-intensity activities and games ranged between 66.4% to 70.4% with most of the misclassifications occurring as either light-intensity activities and games or walking. Recognition of walking and running ranged between 68.5% to 74.4% and 81.5% to 90.0%, respectively. Walking and running instances were most frequently misclassified as light-intensity activities and games.

**Table 2:** Confusion matrix for the hip and wrist random forest physical activity classifiers.

			Prediction				
			SED	L_ACT_G	M_ACT_G	WALK	RUN
Ground Truth	SED	Hip	**82.9**	16.9	0.0	0.0	0.3
		Wrist	**77.4**	21.8	0.6	0.0	0.3
	L_ACT_G	Hip	4.6	**83.9**	5.8	6.9	0.9
		Wrist	7.9	**82.6**	2.3	6.5	0.6
	M_ACT_G	Hip	3.5	14.2	**70.4**	7.0	4.9
		Wrist	5.0	12.0	**66.4**	9.0	3.6
	WALK	Hip	0.0	19.6	4.5	**74.4**	1.5
		Wrist	0.0	23.0	8.5	**68.5**	0.0
	RUN	Hip	0.0	6.7	3.3	0.0	**90.0**
		Wrist	0.0	13.0	3.7	1.9	**81.5**

SED = Sedentary behavior; L_ACT_G = light intensity activities and games; M_ACT_G = moderate-to-vigorous activities and games, WALK = brisk walking; RUN = Running.

Bold numbers represent correct predictions.

### Evaluation in the hold-out sample

**Fig 1:**
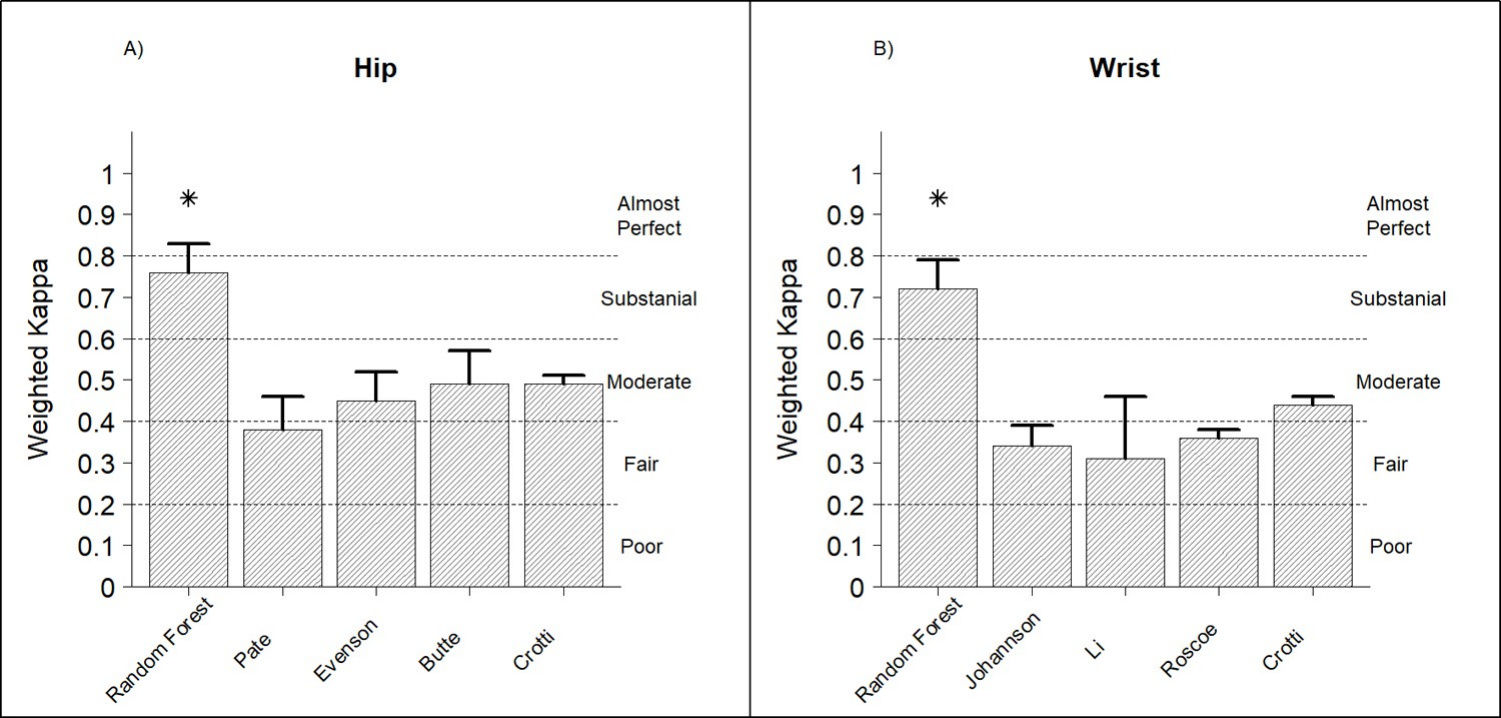
Kappa statistics for the random forest physical activity classifiers and cut-points. Error bars = 95% CI; Asterisk (*) indicates significantly different from all cut points for that placement (P < 0.05).

Fig 1 displays Kappa statistics summarising the agreement between observed and predicted physical activity intensity in the hold out sample. For the hip placement (Fig 1A), the random forest classifier (κ = 0.76) exhibited significantly higher agreement with ground truth physical activity intensity than the cut points (κ = 0.38–0.49). For the wrist placement (Fig 1B), the random forest classifier (κ = 0.72) exhibited significantly higher agreement with ground truth physical activity intensity than the cut points (κ = 0.31–0.44).

**Fig 2:**
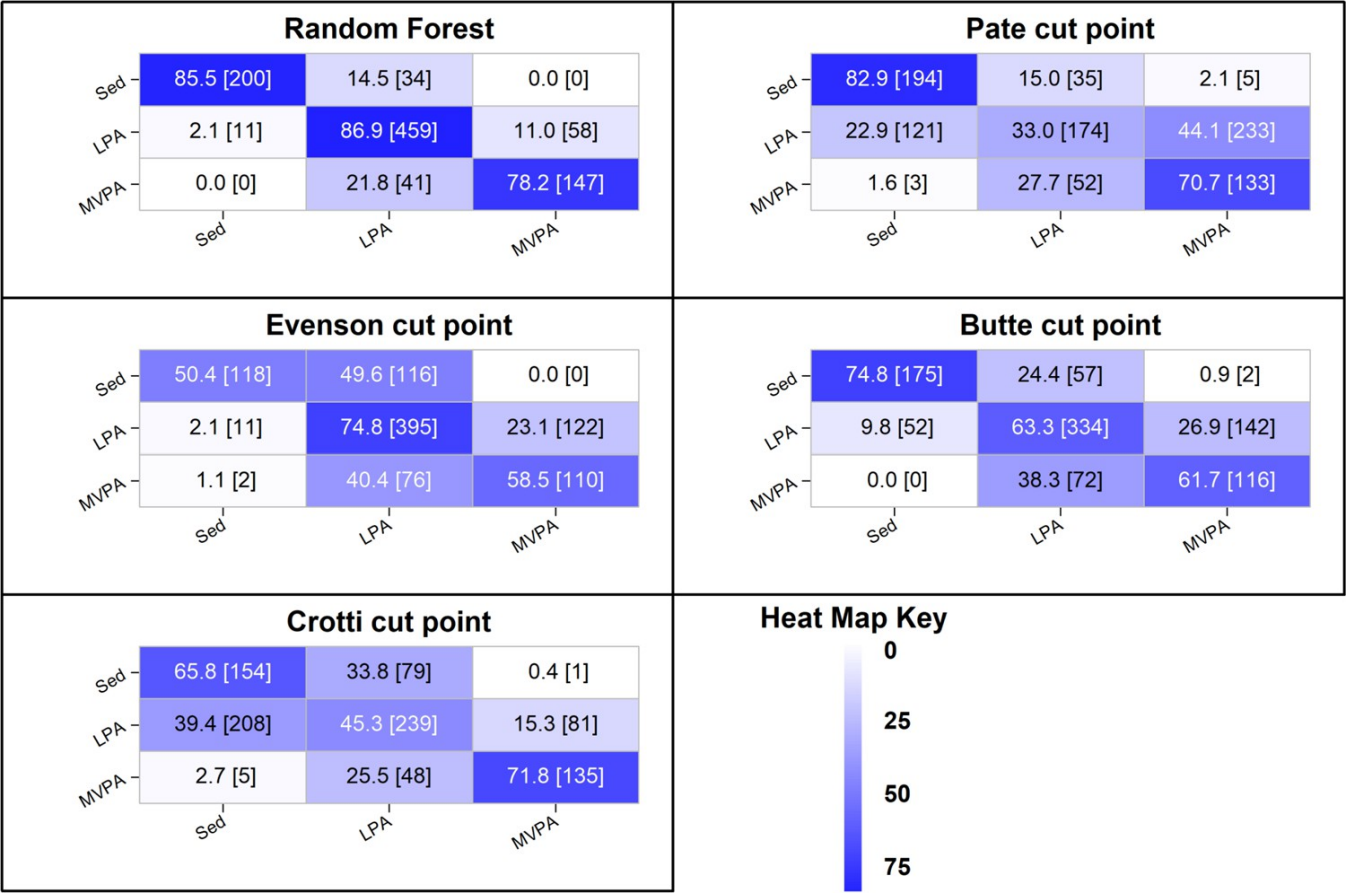
Heat map confusion matrix for the hip placement random forest physical activity classification model and cut-points. Diagonal = correct predictions; Columns = predictions; Rows = ground-truth.

Heat map confusion matrices for classification of physical activity intensity for the hip random forest classifier and cut-points are displayed in Fig 2. For the random forest classifier, recognition of SED and LPA exceeded 85.0% accuracy. Less than 15% of true SED windows were misclassified as LPA, while only 2% of the true LPA windows were misclassified as SED. Eleven percent of true LPA windows were misclassified as MVPA, while no true SED windows were misclassified as MVPA. Recognition accuracy for MVPA was just over 78%, with 22% of true MVPA windows misclassified as LPA. No true MVPA windows were misclassified as SED. Apart from Pate, the cut-points consistently misclassified SED windows as LPA, with 24.0% to 50.0% of true SED windows misclassified as LPA. Recognition of LPA was also generally poor for all cut-points, ranging from 33.0% for Pate to 75.0% for Evenson. For the Evenson, Pate, and Butte cut-points, true LPA windows were most frequently misclassified as MVPA, while the Crotti cut-points misclassified true LPA windows as SED. Recognition of MVPA was poor to modest for all cut-points, ranging from 59.0% (Evenson) to 71.0% (Pate). Most of the true MVPA windows were misclassified as LPA, with very few true MVPA windows misclassified as SED.

**Fig 3:**
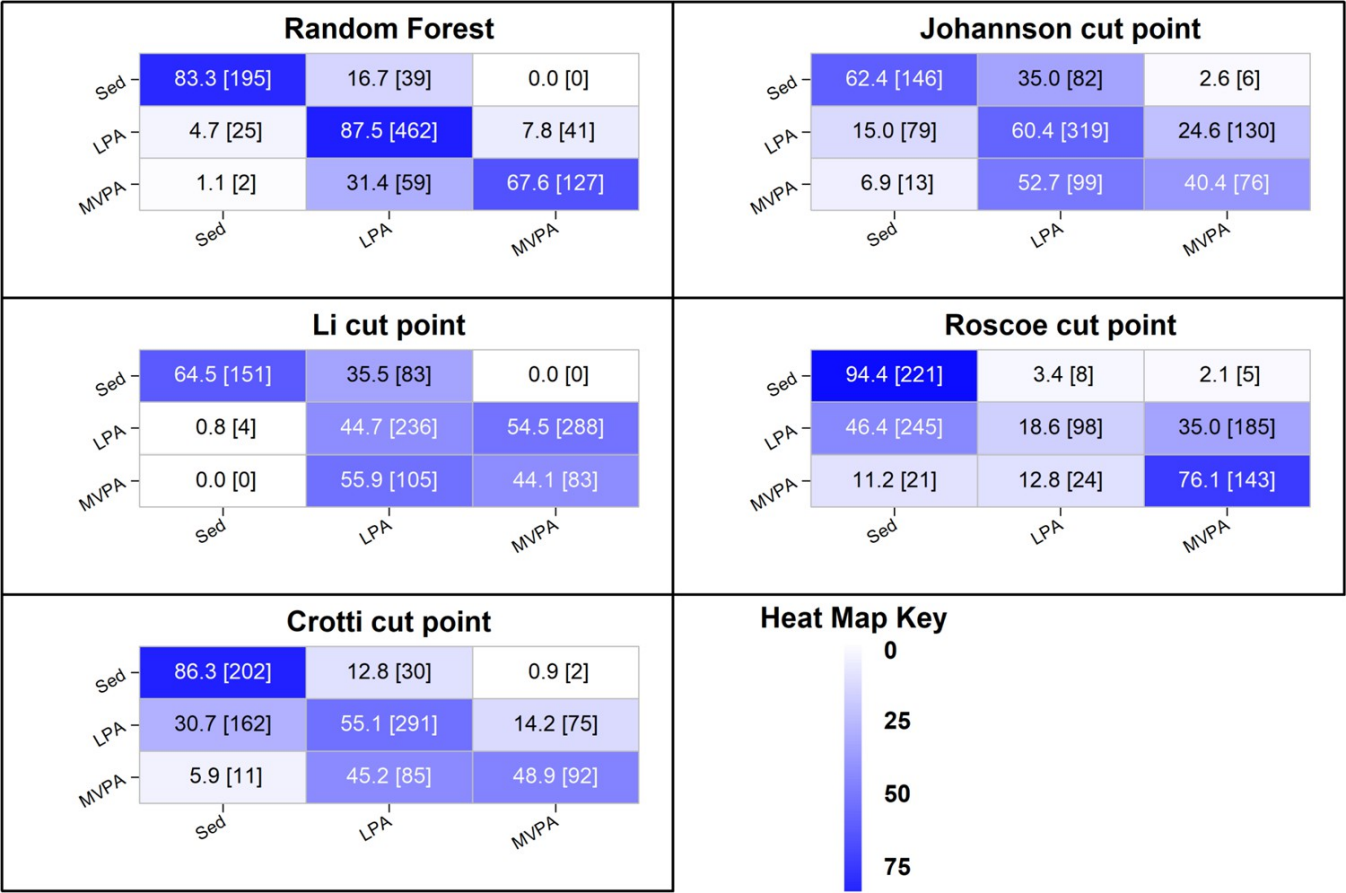
Heat map confusion matrix for the wrist placement free-living classifier cut-points. Diagonal = correct predictions; Columns = predictions; Rows = ground-truth.

Heat map confusion matrices for classification of physical activity intensity for the wrist random forest classifier and cut-points are displayed in Fig 3. For the wrist random forest classifier, recognition of SED and LPA exceeded 80% accuracy. Less than 17% of true SED windows were misclassified as LPA, while less than 5% of true LPA windows were misclassified as SED. Just under 8% of true LPA windows were misclassified as MVPA, while no true SED windows were misclassified as MVPA. Recognition accuracy for MVPA was 68%, with just over 30% of true MVPA windows misclassified as LPA. Only two true MVPA windows (~1%) were misclassified as SED. In contrast, the Johansson and Li cut-points misclassified 35% of true SED windows as LPA, while the Crotti and Roscoe cut-points misclassified between 3% and 13% of true SED windows as LPA. Recognition of LPA was generally poor for all cut-points, ranging from 18.6% (Roscoe) to 60.4% (Johansson). Apart from the Li cut-points, where almost all true LPA windows were misclassified as MVPA, true LPA windows were misclassified as either SED or MVPA. For the Roscoe cut-points, recognition of MVPA was good at 77%; but for the other cut-points, MVPA recognition accuracy was generally poor, ranging from 40.3% (Johansson) to 48.7% (Crotti). True MVPA windows were most frequently misclassified as LPA, except for Roscoe, where true MVPA windows were equally misclassified as SED or LPA.

### Equivalency testing

Table 3 displays means and standard deviations for time in SED, LPA, and MVPA during the free play session based on direct observation, the machine learning classification models, and the different cut-points. On average, children accumulated approximately 6 minutes of sedentary time, 13 minutes of light intensity physical activity, and just under 5 minutes of MVPA.

**Table 3:** Descriptive statistics for time in SED, LPA, and MVPA during the 20-min free play session as determined by direct observation, machine learning classifiers, and intensity-based cut-points.

		Physical Activity Intensity		
		SED	LPA	MVPA
	Direct Observation	5.9 (2.3)	13.2 (3.9)	4.8 (2.8)
Hip				
	Random Forest	5.4 (1.6)	13.4 (3.7)	5.1 (2.9)
	Pate	7.5 (2.0)	6.6 (1.6)	9.8 (5.8)
	Evenson	3.8 (0.9)	14.2 (2.4)	5.9 (3.9)
	Butte	5.3 (0.7)	12.2 (1.8)	6.3 (3.7)
	Crotti	9.8 (2.5)	8.4 (1.8)	5.7 (2.8)
Wrist				
	Random Forest	5.5 (1.8)	14.1 (3.0)	4.3 (2.1)
	Johansson	6.0 (2.0)	11.8 (3.3)	6.1 (3.0)
	Li	1.6 (1.4)	10.0 (3.2)	12.3 (4.3)
	Roscoe	11.1 (2.4)	4.7 (2.6)	8.1 (3.8)
	Crotti	10.0 (2.2)	9.6 (2.8)	4.4 (1.7)

Values represent means (SD).

**Fig 4:**
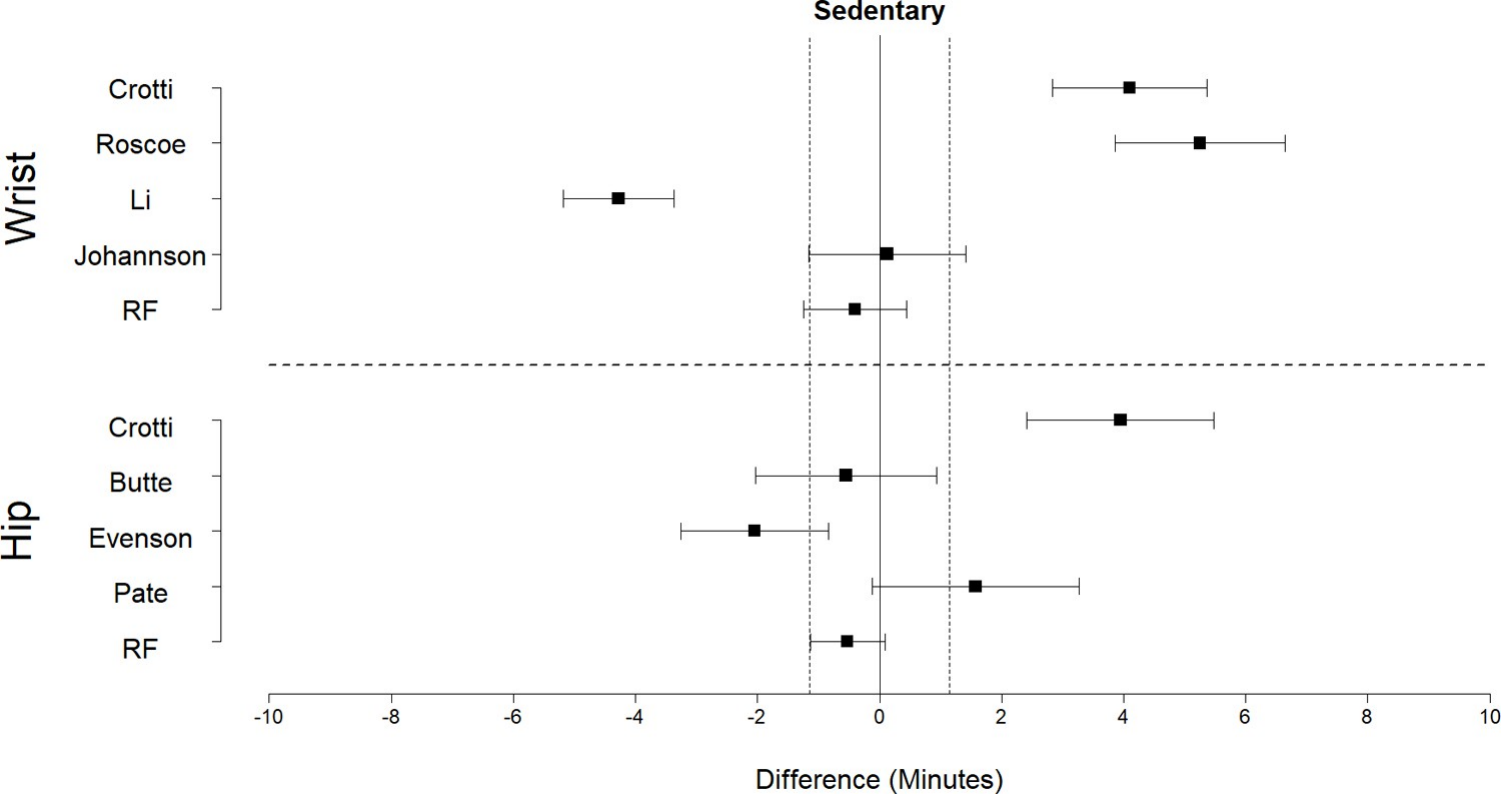
Equivalence plots for the hip and wrist models for time in sedentary activities during the free play session. Difference = predicted time—observed time; Equivalence bounds = ± 1.1 min.

**Fig 5:**
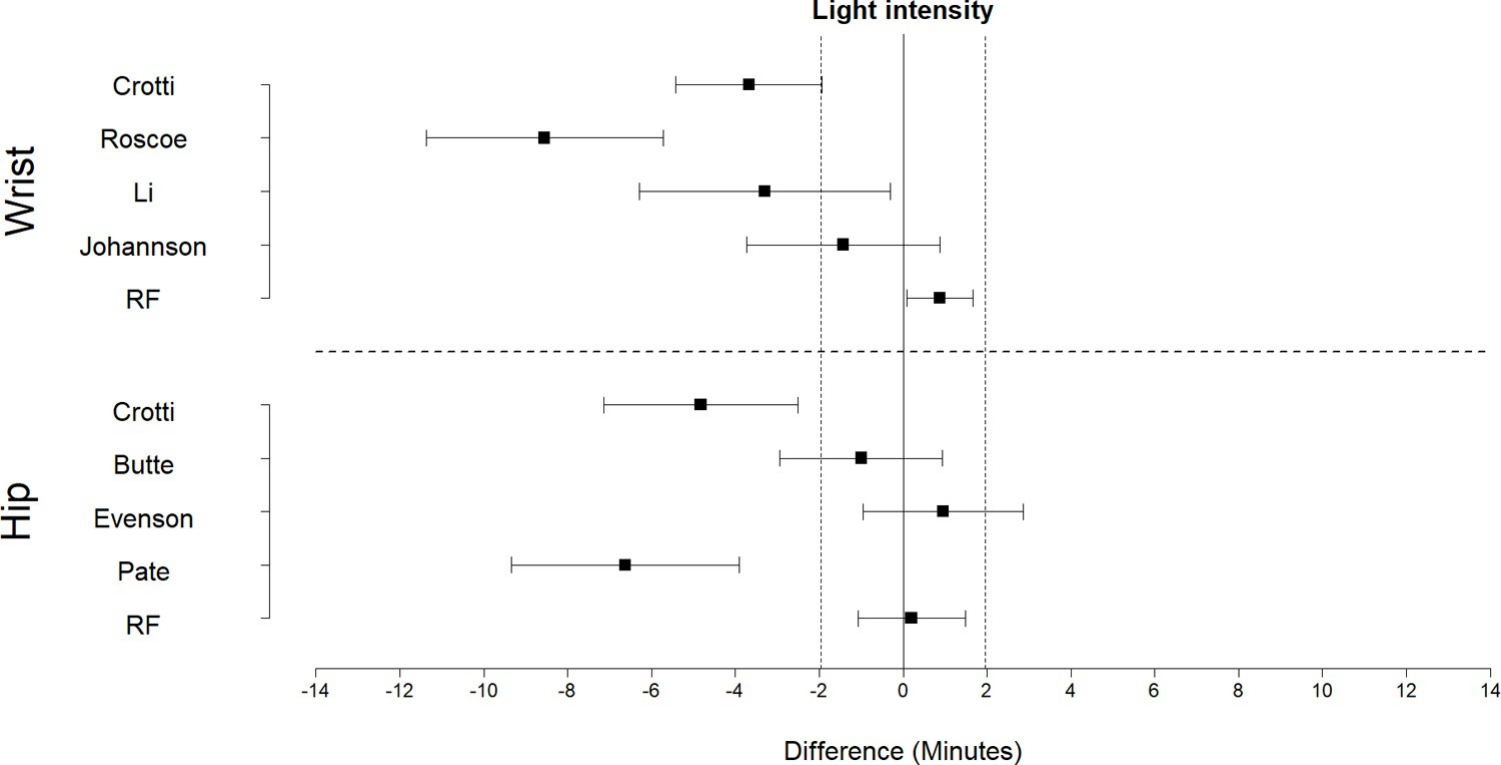
Equivalence plots for hip and wrist models for light-intensity activity during the free play session. Difference = predicted time—observed time; Equivalence bounds = ± 1.9 min.

**Fig 6:**
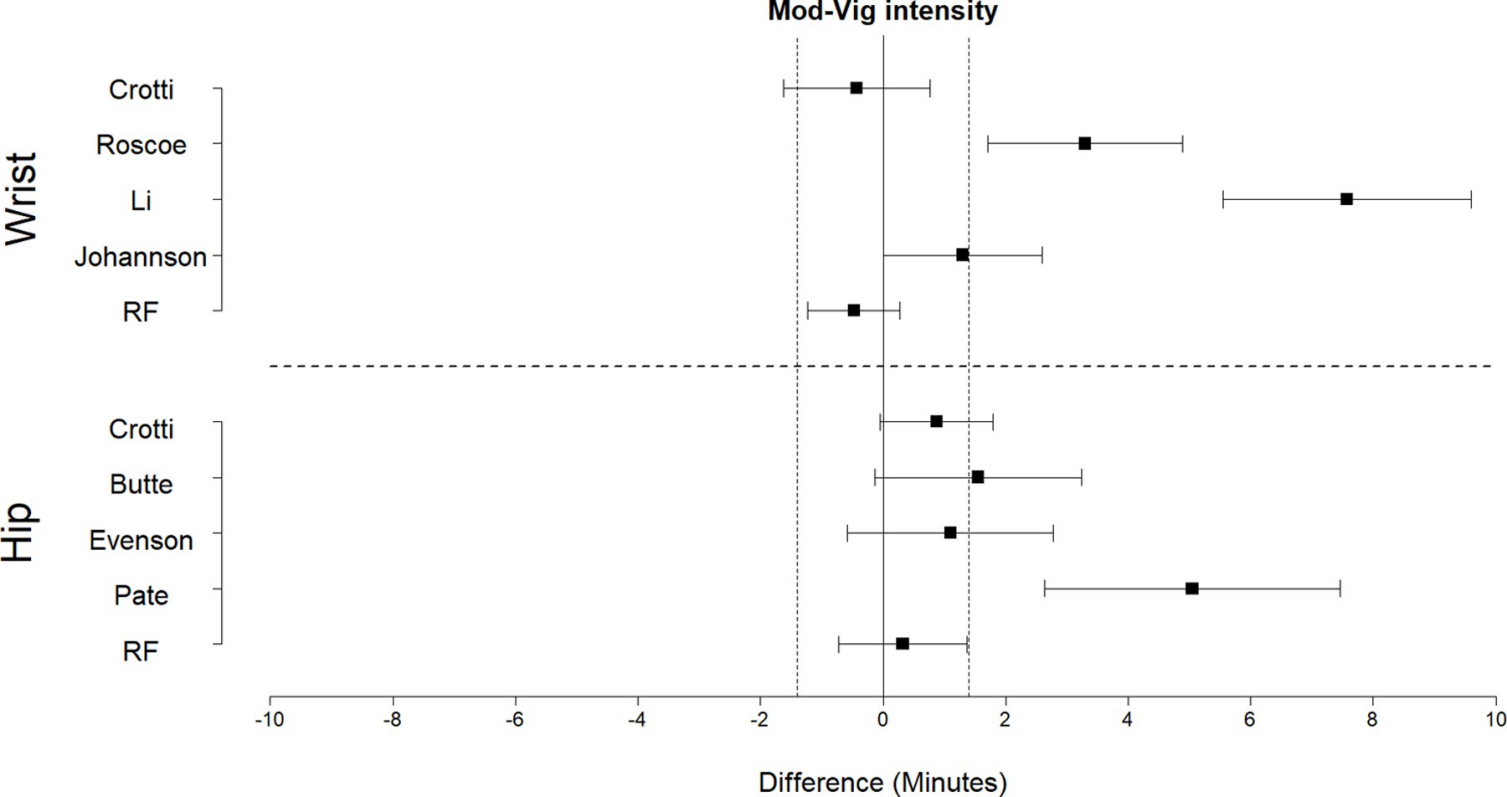
Equivalence plots for hip and wrist models for MVPA during the free play session. Difference = predicted time—observed time; Equivalence bounds = ± 1.4 min.

Equivalence plots for time in SED, LPA, and MVPA predictions are displayed in Figs 4–6, respectively. For time in SED, only the hip random forest classifier exhibited evidence of equivalence to directly observed SED time (t(9) = 1.87, p = 0.047). The wrist random forest classifier, the Butte cut-point for the hip, and the Johansson cut-point for the wrist provided predictions within 0.1 to 0.6 minutes of directly observed SED time; however, the 90% confidence interval exceeded the equivalence bounds.

For time in LPA, the random forest classifier for the hip (t(9) = -2.51, p = 0.017) and wrist (t(9) = -2.48, p = 0.017) exhibited evidence of equivalence. The cut-points consistently underestimated time in LPA, and none exhibited evidence of equivalence. The Butte cut-point for the hip and the Johansson cut-point for the wrist provided predictions within 1.0 to 1.2 minutes of observed time in LPA; however, the 90% confidence interval exceeded the equivalence bounds.

For time in MVPA, the random forest classifiers for the hip (t(9) = -1.87, p = 0.047) and wrist (t(9) = 2.25, p = 0.025) exhibited evidence of equivalence to directly observed time in MVPA. In comparison, the cut-points consistently overestimated MVPA, and none exhibited evidence of equivalence. The Evenson and Crotti cut-points for the hip and the Crotti cut-point for the wrist provided predictions within 0.4 to 1.1 minutes of directly observed time in MVPA; however, the 90% confidence interval exceeded the equivalence bounds.

## Discussion

The current study compared the accuracy of physical activity intensity predictions provided by machine learning classification models to those provided by previously published cut-points for preschool-aged children. Under true free-living conditions, machine learned random forest classification models for the hip and wrist exhibited significantly higher agreement with ground-truth physical activity intensity than cut-point methods. Moreover, the machine learning models exhibited evidence of equivalence with directly observed time in sedentary activity, LPA, and MVPA during the ad libitum play sessions. In contrast, cut-points methods consistently overestimated time in sedentary behaviour, underestimated time in LPA, and overestimated time MVPA. None of the cut-points exhibited evidence of equivalence. The results demonstrate the relative advantage of machine learning methods over traditional threshold-based approaches for quantifying physical activity behaviour in young children.

The lower accuracy of the cut-points was attributable to, in large part, the consistent misclassification of LPA as either SED or MVPA. In theory, threshold methods seek to identify a cut-off value that delineates adjacent levels of physical activity intensity with optimal sensitivity and specificity. That is, the identified cut-point minimizes the number of false negatives (FN) and false positives (FP). However, in practice, there is always a trade-off between sensitivity and specificity [42]; and the cut-points derived in calibration studies inevitably sacrifice specificity for sensitivity and vice versa. The effects of this trade off on classification accuracy were illustrated in the heat map confusion matrices shown in Figs 2 and 3. For detection of SED windows, thresholds relatively high in magnitude (Roscoe, Crotti, Pate) sacrificed specificity for sensitivity. A relatively small percentage of true SED windows were misclassified as LPA (FN); however, a relatively large percentage of true LPA windows were misclassified as SED (FP). Conversely, SED thresholds relatively low in magnitude (Evenson, Li, Johansson), sacrificed sensitivity for specificity. A relatively small percentage of true LPA windows were misclassified as SED (FP) but a relatively large percentage of true SED windows were misclassified as LPA (FN). The pattern was reversed for detection of MVPA windows. Relatively low thresholds for MVPA (Roscoe, Pate) sacrificed specificity for sensitivity. A relatively small percentage of true MVPA windows were misclassified as LPA (FN), but a relatively large percentage of true LPA windows were misclassified as MVPA (FP). Conversely, relatively high thresholds for MVPA (Evenson, Johansson, Crotti wrist) sacrificed sensitivity for specificity. A relatively small percentage of true LPA windows were misclassified as MVPA (FP), but a relatively large percentage of true MVPA windows were misclassified as LPA (FN). As a result of these trade-offs, all eight sets of cut-points detected LPA with poor sensitivity and/or specificity, resulting in significant misclassifications errors for SED, LPA, and MVPA. Unlike cut-point methods, machine learning physical activity classification models do not predict physical activity intensity category from a single parameter (magnitude of acceleration). They are trained to recognize patterns and regularities in the data and make predictions of physical activity type or class. As such, the trade-off between sensitivity and specificity associated with identifying a cut-off value does not apply.

The random forest PA classification models for the hip and wrist exhibited significantly better agreement with directly observed physical activity intensity and provided more accurate predictions of time in intensity categories than the cut-point methods. We therefore recommend that researchers discontinue the use of cut-points and implement currently available physical activity classification models for preschool-aged children [19]. Nevertheless, it is important to acknowledge that the random forest classification models were not without error. In the leave-one-out cross-validation, 17% to 22% of true sedentary windows were misclassified as light-intensity activities and games, 20% to 23% of true walking windows were misclassified as light-intensity activities and games, and 12% to 14% of true moderate-to-vigorous activities and games windows were misclassified at light-intensity activities and games. When the five physical activity classes were mapped to the three physical activity intensity categories, these errors resulted in some misclassification of MVPA as LPA. These misclassifications can be explained, at least in part, by the difficulty associated with monitoring physical activity with a single accelerometer worn on the wrist or hip, for which the output recorded during some physical activity types may not be strongly predictive of physical activity intensity. Ahmadi et al. [25] used a customized direct observation system to gain insight into why machine learning models misclassified certain classes of physical activity. The results indicated that sedentary behaviours were more likely to be misclassified as light-intensity activities and games if they were performed with significant upper body movement. Moderate-to-vigorous activities and games were more likely to be misclassified as light-intensity activities and games when children engaged in activities that required high levels of energy expenditure but relatively little movement, such as climbing on fixed equipment in the playground. Walking was frequently misclassified as light-intensity activities and games, because, under true free-living conditions, children often walked in a slow and meandering manner consistent with the concept of "pottering around". Therefore, although the machine learning models evaluated in this study provided more accurate estimates of physical activity intensity than cut-point methods and are available for immediate deployment, research efforts to refine and improve the accuracy machine learning data processing methods must continue. Future studies could explore the utility of feature fusion using data from other sensor types, such as heart rate monitors and barometric pressure sensors. Existing ML models could be periodically "updated" and retrained using free-living training data collected over extended time periods to capture a wider range of activities performed under different contexts. Finally, investigators should explore the performance of new and emerging supervised learning algorithms such as custom ensembles, gradient boosting models as well as explore deep learning approaches [21].

The current study had several strengths. To our knowledge, it is first study to directly compare the accuracy of machine learning activity classifiers and published cut points in preschool aged children under true free-living conditions. The study design allowed for children’s physical activity behaviour to be assessed across a wide variety of activities across the full spectrum of physical activity intensities in a variety of real-world settings. Ground truth physical activity intensity was quantified using a validated direct observation system that has been widely used in studies of preschool children [43, 44]. Each play-session was video-recorded to ensure valid and reliable physical activity intensity coding and annotation. The study included accelerometers worn on the hip and wrist, which are the most common wear locations for studies involving preschool-aged children, and all previously published cut points for this age group were evaluated. This added to the comprehensiveness of the evaluations and outcomes.

There were also several limitations that warrant consideration. The sample of 31 children was relatively small, with 21 children randomly assigned to the training sample to develop the machine learning models and 10 assigned to the hold out sample to compare the accuracy of the machine learning models and cut-point methods. In addition, due to the demands of the data collection and direct observation coding protocols, the duration of the play-session was limited to approximately 20 minutes. This reflects the trade-off between obtaining quality ground-truth measures and feasibility of data collection in studies involving young children. However, it is important to note that the sample size and duration of the free play session were more than adequate to address the aims of the study. The free play sessions provided a wide range of activities representative of preschool-aged children’s physical activity behaviours. Across the 31 sessions, we observed 22 different activities ranging in physical activity intensity, tempo, movement characteristics. Moreover, with a sampling rate of 100 Hz, each play session generated approximately 120,000 data instances and more than 300 15-sec windows for each placement site. Thus, the "calibration" sample of 21 children provided more than 6,000 fully annotated 15-second windows for model development and testing, while the 10 children in the hold out sample provided more than 3000 fully annotated 15-sec windows for analysis and comparison.

## Conclusions

In conclusion, under true free-living conditions, machine learning physical activity classification models for preschool-aged children exhibited significantly better recognition of physical activity intensity than cut-point methods. The results demonstrate the relative advantage of machine learning methods over traditional threshold-based approaches and adds to a growing evidence base supporting the feasibility and accuracy of machine learning accelerometer data processing methods. We encourage researchers to use the random forest physical activity classification models evaluated in this study. The final models, annotated datasets, and code for implementation in the open-source R software environment is available at: https://github.com/QUTcparg/Preschool-ML-vs-CP. Future studies should expand upon the findings of current study and compare the performance of machine learning classification models and cut points in free-living pre-adolescent and adolescent children. Moving forward, to increase the uptake of machine learning accelerometer data processing methods, user-friendly open-source software that extracts the necessary acceleration features and applies previously validated machine learning models should be made available for health researchers.

## Supporting information

S1 File Information about free play sessions. (DOCX)
