# Supplementary material for: Device-based measurement of physical activity in pre-schoolers: Comparison of machine learning and cut point methods
Source: PLoS One. 2022 Apr 13;17(4):e0266970. doi: 10.1371/journal.pone.0266970 (PMC9007358; doi:10.1371/journal.pone.0266970)
Supplement: S1 File — (DOCX) [file pone.0266970.s001.docx]

**Device-based measurement of physical activity in pre-schoolers: comparison of machine learning and cut point methods**

**Supplementary Document**

| **Page** | **Item** |
| --- | --- |
| 1 | Supplemental Table 1: Duration of activities during free play sessions |
| 2 | Supplemental Text 1: Description and descriptive statistics of accelerometer features |

Supplemental Table 1: Duration of activities during free play sessions

|  |  | **Duration (s)** | |
| --- | --- | --- | --- |
| **Activity Type** | **Participants (N)** | **Median** | **Range** |
| **Active Play Session** | **31** | **1230.4** | **1185.2 - 1260.7** |
|  |  |  |  |
| Sit still | 28 | 95.2 | 37.3 - 264.6 |
| Sit w/upper body movement | 28 | 189.2 | 7.0 - 435.4 |
| Stand still | 31 | 80.1 | 6.4 - 174.1 |
| Stand w/upper body movement | 31 | 167.0 | 75.5 - 378.1 |
| Crawl | 10 | 35.7 | 12.8 - 54.3 |
| Up/down stairs | 9 | 35.6 | 10.0 - 44.2 |
| Floor games | 8 | 53.8 | 8.3 - 89.9 |
| Stand and kick | 11 | 22.3 | 9.4 - 38.6 |
| Slide | 11 | 38.4 | 6.9 - 62.1 |
| Climb (low intensity) | 23 | 119.2 | 7.4 - 401.4 |
| Dribbling | 8 | 151.1 | 19.3 - 257.9 |
| Side gallop | 10 | 92.7 | 3.4 - 144.3 |
| Jump/hop/leap | 31 | 60.4 | 5.2 - 187.3 |
| Ride a bike | 4 | 20.3 | 9.2 - 31.3 |
| Ride a scooter | 1 | 323.7 | 323.7 |
| Playground ride | 13 | 52.9 | 6.3 - 112.0 |
| Climb (high intensity) | 6 | 44.9 | 7.0 - 90.7 |
| Walk slow/stroll | 27 | 82.3 | 57.0 - 208.6 |
| Walk brisk | 21 | 58.5 | 20.7 - 194.4 |
| Walk and hold object | 15 | 35.3 | 4.9 - 112.7 |
| Run | 26 | 89.3 | 31.2 - 167.7 |
| Run and hold object | 8 | 34.4 | 5.5 - 53.8 |
| out of view | 6 | 12.8 | 8.4 - 69.1 |

**Supplemental Text 1: Description and descriptive statistics of accelerometer features**

Features were extracted from 15 second non-overlapping sliding windows. Time-domain features included: mean, standard deviation, coefficient of variation, 10^th^ percentile, 25^th^ percentile, 50^th^ percentile, 75^th^ percentile, 90^th^ percentile, skewness, kurtosis, maximum, minimum, peak to peak amplitude, median crossings, sum, mean absolute deviation, signal power, lag-1 autocorrelation, log energy, interquartile range, XY cross-axis correlation, XZ cross-axis correlation, and YZ cross-axis correlation were simple time-domain features extracted from the vector magnitude of the accelerometer signal.

In addition to these simple time domain features, fast Fourier transformation (FFT) was used to extract frequency domain features of the acceleration signal between 0.25 and 5.0 Hz. FFT gives a set of basis coefficients that represent the amplitudes of the frequency components of the signal and distribution of the signal energy. The dominant frequency and its magnitude were also extracted. The frequency with highest FFT magnitude was considered as the dominant frequency.

Below is the median and range of the feature values from the 31 participants:

| **Feature** | **Hip** | **Wrist** |
| --- | --- | --- |
| Mean | 1.1 [1.0, 2.4] | 1.2 [1.0, 4.6] |
| Standard deviation | 0.1 [0.0, 0.8] | 0.2 [0.0, 1.6] |
| Coefficient of variation | 0.1 [0.0, 0.7] | 0.2 [0.0, 0.8] |
| 10^th^ percentile | 0.9 [0.3, 1.0] | 0.9 [0.3, 1.0] |
| 25^th^ percentile | 1.0 [0.5, 1.0] | 1.0 [0.5, 1.5] |
| 50^th^ percentile | 1.0 [0.9, 1.5] | 1.0 [0.9, 2.2] |
| 75^th^ percentile | 1.0 [1.0, 2.1] | 1.1 [1.0, 3.1] |
| 90^th^ percentile | 1.1 [1.0, 2.4] | 1.2 [1.0, 4.6] |
| skewness | 1.4 [-3.4, 14.3] | 1.8 [-2.7, 16.4] |
| kurtosis | 8.7 [-1.5, 276.8] | 9.5 [-1.4, 343.9] |
| maximum | 1.9 [1.0, 8.4] | 2.6 [1.0, 9.4] |
| minimum | 0.6 [0.0, 1.0] | 0.4 [0.0, 1.0] |
| Peak to peak | 1.3 [0.0, 8.4] | 2.2 [0.0, 9.2] |
| Median crossings | 178 [23, 502] | 139 [22, 556] |
| Sum | 1,509 [1,483, 2,062 | 1,548 [1,458, 3,738] |
| Mean absolute deviation | 0.0 [0.0, 1.2] | 0.1 [0.0, 1.3] |
| Signal power | 1,539 [1,467, 3,778] | 1,668 [1,417, 12,358] |
| Lag-1 autocorrelation | 0.9 [0.1, 1.0] | 0.9 [0.4, 1.0] |
| Log energy | -7.2 [-203.7, 378.6] | 42.2 [-161.1, 2,256.8] |
| Interquartile range | 0.1 [0.0, 1.6] | 0.2 [0.0, 2.1] |
| XY | -0.1 [-1.0, 0.8] | -0.2 [-1.0, 1.0] |
| XZ | 0.1 [-0.9, 1.0] | 0.1 [-0.9, 1.0] |
| YZ | 0.0 [-1.0, 1.0] | 0.1 [-0.9, 1.0] |
| Dominant Frequency | 2.5 [0.3, 5.0] | 1.9 [0.3, 5.0] |
| Magnitude of dominant frequency | 0.3 [0.0, 0.8] | 0.6 [0.0, 0.9] |
